# Predictive Value of Eosinophil Count on COVID-19 Disease Progression and Outcomes, a Retrospective Study of Leishenshan Hospital in Wuhan, China

**DOI:** 10.1177/08850666211037326

**Published:** 2021-09-22

**Authors:** Wei Xuan, Xuliang Jiang, Lili Huang, Shuting Pan, Caiyang Chen, Xiao Zhang, Hui Zhu, Song Zhang, Weifeng Yu, Zhiyong Peng, Diansan Su

**Affiliations:** 1 Renji Hospital, 71140 Shanghai Jiaotong University, Shanghai, China; 2 Clinical Center for Investigation, Renji Hospital, Shanghai Jiaotong University, Shanghai, China; 3 Zhongnan Hospital of Wuhan University, Wuhan, China

**Keywords:** asthma, coronavirus, COVID-19, eosinophil, leukocyte

## Abstract

**Background:**

The potential protective role of eosinophils in the COVID-19 pandemic has aroused great interest, given their potential virus clearance function and the infection resistance of asthma patients to this coronavirus. However, it is unknown whether eosinophil counts could serve as a predictor of the severity of COVID-19.

**Methods:**

A total of 1004 patients with confirmed COVID-19 who were admitted to Leishenshan Hospital in Wuhan, China, were enrolled in this study, including 905 patients in the general ward and 99 patients in the intensive care unit (ICU). We reviewed their medical data to analyze the association between eosinophils and ICU admission and death.

**Results:**

Of our 1004 patients with COVID-19, low eosinophil counts/ratios were observed in severe cases. After adjusting for confounders that could have affected the outcome, we found that eosinophil counts might not be a predictor of ICU admission. In 99 ICU patients, 58 of whom survived and 41 of whom died, low eosinophil level was an indicator of death in severe COVID-19 patients with a cutoff value of 0.04 × 10^9^/L, which had an area under the curve of 0.665 (95% CI = 1.089-17.839; *P* = .045) with sensitivity and specificity of 0.569 and 0.7317, respectively.

**Conclusion:**

Our research revealed that a low eosinophil level is a predictor of death in ICU patients rather than a cause of ICU admission.

## Introduction

A newly identified coronavirus, COVID-19, has caused unexpected prevalence of respiratory disease for over half a year. The World Health Organization (WHO) pronounced COVID-19 a pandemic disease on March 11, 2020.^1^ As of the day we completed this article, according to data from the WHO, there have been more than 100 million confirmed cases of COVID-19, causing 2.3 million deaths worldwide.

Given that 14% of patients have developed severe cases of the disease, efforts have been made to clarify the underlying infectious mechanisms and risk factors to reduce mortality.^2,3^ Owing to delayed virus clearance and a severe cytokine storm, severe acute respiratory distress syndrome and multiple organ failure accounted for the deterioration of most severe patients.^4^ According to previous studies,^5-7^ many factors have been proven to be predictive of severe cases, such as older age, male sex, hypertension, diabetes, excessive cytokines, coronary heart disease, and lymphocytopenia. However, many cases develop to severe status without these risk factors. Thus, identifying more risk factors is warranted.

Eosinophils are one of the less common blood leukocytes,^8^ and they can be used to identify and predict the outcome of infectious diseases.^9^ Eosinophils also play a central role in allergic disease,^10^ in which levels are increased in the pathological processes of asthma and allergy.^11^ Compared with other comorbidities, fewer patients with asthma have been found in the COVID-19 pandemic, which could be partly attributable to the virus-resistant function of eosinophils.^12^ More than this, eosinopenia on admission may be a reliable and convenient early diagnostic marker for COVID-19 infection before nucleic acid test results.^13^ Although eosinophil levels might have vital clinical relevance to COVID-19 recovery because of their pro-inflammation and potential virus elimination properties, it is unknown whether eosinophil counts could serve as a predictor of the severity of COVID-19.^14^ Given that a larger sample of clinical patients with COVID-19 was warranted,^15^ we conducted a retrospective study involving 1004 patients with COVID-19 to analyze the association between eosinophils and intensive care unit (ICU) admission and death.

## Methods

### Study Design and Patients

We performed a single-center, retrospective review of patients admitted to Leishenshan Hospital in Wuhan, China, one of the hospitals designated to treat patients with COVID-19. A total of 1004 patients with confirmed COVID-19 were enrolled in this study, including 905 patients in the general ward and 99 patients in the ICU during February 8 to April 15, 2020. All patients with COVID-19 pneumonia in this study were diagnosed according to the interim guidance for diagnosis and treatment provided by the National Health Commission of China and the WHO. All patients tested positive for COVID-19 by analyzing body fluid samples using quantitative reverse transcription-polymerase chain reaction (qRT-PCR). Patients’ data were obtained by reviewing electronic medical records. This study was approved by the Ethics Commission of Renji Hospital (Ethical Committee approval number: KY2020-037). Informed consent was waived and approved by the Ethics Commission of Renji Hospital because of its retrospective nature.

### Data Collection

The medical records of all patients were independently obtained by the authors, who worked for the Critical Care Medicine Department of Leishenshan Hospital at that time, and laboratory data were reviewed from electronic medical records. All body fluid samples were analyzed and diagnosed by local health authorities as recommended by the Chinese Center for Disease Control and Prevention (CDC) and by using qRT-PCR with the CDC-approved process. The medical information collected included age, sex, laboratory values, and chronic disease histories (eg, cardiac disease, cerebrovascular disease, pulmonary disease, malignancy, neurological disease, and diabetes).

### Statistical Analysis

For the baseline characteristics, categorical variables were summarized as frequencies and percentages, and continuous variables were described using median and interquartile range (IQR). Patients with ICU admission were matched with those without ICU admission at a 1:2 ratio based on their propensity scores, which were developed by considering variables that could potentially affect the outcome. The matching performance was assessed with the Kruskal–Wallis rank sum test, in which a *P* value less than .05 was selected for adjustment in the following analysis.

To evaluate the difference in eosinophils between ICU and non-ICU patients, the optimal eosinophil cutoff point was determined based on receiver operating characteristic (ROC) curve analysis, and then, multivariate logistic regression was used, of which the results would be presented using an odds ratio (OR) with a 95% confidence interval (CI) for each covariate.

Prognostic analyses of ICU patients were conducted using logistic regression, as well as when in-hospital mortality was taken as the outcome variable, and eosinophils and other confounders were covariates. Covariates were selected by utilizing stepwise regression using the Akaike information criterion.

Statistical analyses were performed using R (version 4.0.0), and *P* < .05 was considered statistically significant.

## Results

### Demographics and Baseline Characteristics of Patients with COVID-19

The baseline characteristics of all hospitalized patients are shown in Table 1. The percentage of male patients in the ICU was 68.7% compared to 47.1% in the general ward (*P *< .001). The patients’ median age was 60 years (IQR, 49-69), with the median age of the patients in the general ward (58; IQR, 47-68) younger than that of those in the ICU (69; IQR, 62-80) (*P* < .001). Among all patients, the most common comorbidities were hypertension (27.8%), followed by diabetes (11.8%), cardiovascular disease (8.7%), pulmonary disease (3.5%), stroke (3.0%), chronic renal insufficiency (2.7%), and chronic hepatitis and cirrhosis (1.9%). Compared with the general ward, the comorbidities mentioned above were more commonly found in the ICU patients, whereas no significant difference was found in cancer rates between the 2 groups. In terms of laboratory results, higher counts and percentages of eosinophils and lymphocytes were shown in the general ward patients compared with those in the ICU. Lower white blood cell (WBC) counts, neutrophil counts, and percentages were reported in the general ward patients (*P *< .001) (Table 1).

**Table 1.** Demographics, Baseline Characteristics, and Laboratory Results of all Patients with COVID-19.

Characteristics	All patients (n = 1004)	General ward (n = 905)	ICU (n = 99)	*P* value
Male, n (%)	494 (49.2%)	426 (47.1%)	68 (68.7%)	<.001
Age, median (range)	60 (49-69)	58 (47-68)	69 (62-80)	<.001
Any comorbidity, n (%)				
Hypertension, n (%)	280 (27.8%)	235 (25.9%)	45 (45.5%)	<.001
Diabetes, n (%)	119 (11.8%)	101 (11.1%)	18 (18.2%)	.04
Cardiovascular disease, n (%)	88 (8.7%)	67 (7.4%)	21 (21.2%)	<.001
Pulmonary disease, n (%)	35 (3.5%)	27 (3.0%)	8 (8.1%)	.019
Stroke, n (%)	30 (3.0%)	15 (1.7%)	15 (15.2%)	<.001
Malignancy, n (%)	13 (1.3%)	11 (1.2%)	2 (2%)	.838
Chronic renal insufficiency, n (%)	27 (2.7%)	19 (2.1%)	8 (8.1%)	.002
Chronic hepatitis and cirrhosis, n (%)	36 (1.9%)	26 (2.9%)	10 (10.1%)	.001
White blood cell count (3.5-9.5 × 10^9^/L)	5.68 (4.7-7.09)	5.56 (4.63-6.78)	9.53 (6.15-11.82)	<.001
Neutrophil count (1.8-6.3 × 10^9^/L)	3.31 (2.55-4.48)	3.16 (2.51-4.12)	7.48 (4.48-10.48)	<.001
Neutrophil percentage (40%-75%)	58.9 (52.35-67.35)	57.95 (51.6-63.92)	84.3 (73.5-89.7)	<.001
Lymphocyte count (1.1-3.2 × 10^9^/L)	1.56 (1.16-1.94)	1.61 (1.26-1.99)	0.79 (0.5-1.2)	<.001
Lymphocyte percentage (20%-50%)	28.1 (21.1-34.1)	29.3 (23.7-34.9)	8.4 (5.5-15.6)	<.001
Eosinophil count (0.02-0.52 × 10^9^/L)	0.1 (0.06-0.17)	0.11 (0.06-0.18)	0.03 (0.0-0.13)	<.001
Eosinophil percentage (0.4%-8%)	1.8 (1-3)	1.9 (1.1-3.12)	0.2 (0-1.5)	<.001

Data are the median (IQR) or n/N (%). The normal range of laboratory test were listed in brackets in the first column. *P* values comparing general ward cases and ICU cases are from χ^2^, Fisher’s exact test, or Mann–Whitney *U* test. The frequencies of categorical variables were compared using the χ^2^ and Fisher’s exact test as appropriate.

Abbreviations: ICU, intensive care unit; IQR, interquartile range.

### Propensity Score Matching Results Showed That Circulating Eosinophil Count was not an Indicator for ICU Admission

**Figure 1.**
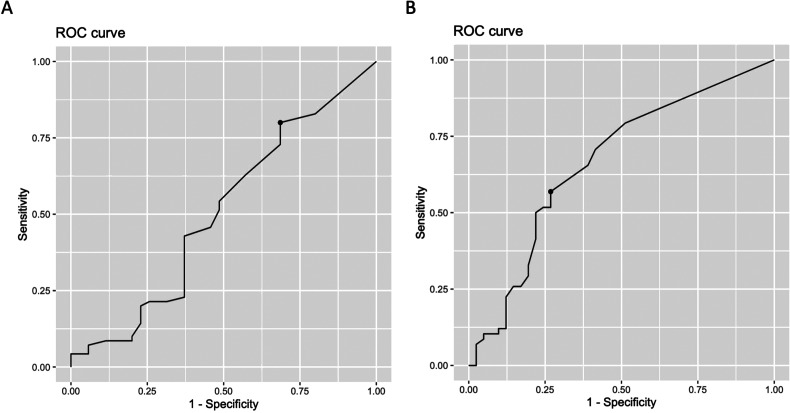
ROC curve analysis of predictive value of EOS for ICU admission and death of ICU patients. (A) EOS counts had AUC of 0.504 and the cutoff value was 0.02 × 10^9^/L for prediction of ICU admission, the sensitivity was 0.800 and specificity was 0.3143. (B) EOS counts had AUC of 0.665 and the cutoff value was 0.04 × 10^9^/L for prediction of death of ICU patients, the sensitivity was 0.569 and specificity was 0.7317.

Abbreviations: ROC, receiver operating characteristic; ICU, intensive care unit; AUC, area under the curve.

In order to evaluate the role of eosinophil count in ICU admission after balancing the baseline characteristics of the patients in the general ward and ICU, we conducted propensity score matching of 70 non-ICU patients and 35 ICU patients. The matched baseline characteristics are described in Supplemental Table 1, in which the differences between groups are shown. Age, sex, and other comorbidities (eg, coronary heart disease and diabetes) that could affect the outcome were balanced between the general ward and ICU patients. Other risk factors, including hypertension, C-reactive protein (CRP), urea, glucose, D-dimer, activated partial thromboplastin time (APTT), and procalcitonin, whose *P* values were less than .05 were subsequently selected for adjustment. The predictive value of eosinophil counts was evaluated using ROC curves, and the optimal eosinophil cutoff point was 0.02 × 10^9^/L, which maximized the sum of sensitivity and specificity, leading to a 0.504 area under the curve (AUC) (Figure 1A). However, there was no significant difference between the effect of eosinophil count <0.02 × 10^9^/L (n = 25) and eosinophil count ≥0.02 × 10^9^/L (n = 80) on ICU admission (OR, 1.216; 95% CI, 0.827-5.610) after considering other confounders. Only CRP played an important role in predicting the ICU admission of severe patients (OR, 1.013; 95% CI, 1.001-1.026) (Table 2).

**Table 2.** Multivariable Logistic Regression ORs (95%CI) for ICU Admission.

Covariates	Odds ratio	95% CI	*P* value
Hypertension	2.149	(0.827, 5.61)	.114
CRP	1.013	(1.001, 1.026)	.039
Urea	0.997	(0.973, 1.02)	.816
GLU	1.033	(0.946, 1.143)	.496
D_dimer	1.01	(0.978, 1.041)	.491
APTT	1.001	(0.983, 1.016)	.895
PCT	1.02	(0.716, 1.457)	.906
EOS count			.732
≥0.02 × 10^9^/L (n = 25)	1		
<0.02 × 10^9^/L (n = 80)	1.216	(0.38, 3.659)	

Abbreviations: CRP, C-reactive protein; GLU, glucose; APTT, activated partial thromboplastin time; PCT, procalcitonin; OR, odds ratio; CI, confidence interval; ICU, intensive care unit.

### Demographics, Baseline Characteristics, and Laboratory Results of ICU Patients with COVID-19

The baseline characteristics of the ICU patients on admission are shown in Table 3. There was no significant difference in sex between those who survived and those who died. The median age of the ICU patients was 69.0 years (IQR, 62.0-80.0), with the median age of those who died (73.0; IQR, 65.0-81.0) older than those who survived (66.0; IQR, 59.5-77.5) (*P*  =  .048). Most of the ICU patients were male (68.7%), the male-to-female ratio was greater than 2:1, and most deaths (65.9%) were of male patients. Among all ICU patients, nearly half (45.5%) had hypertension, and some had other comorbidities, but there was no significant difference between the survival and death cases. The most frequent clinical symptoms were hypertension (45.5%), cardiovascular disease (21.2%), diabetes (18.2%), stroke (15.2%), chronic hepatitis and cirrhosis (10.1%), pulmonary disease (8.1%), chronic renal insufficiency (8.1%), and malignancy (2%).

**Table 3.** Demographics, Baseline Characteristics, and Laboratory Results of Patients with COVID-19 in ICU.

Characteristics	All ICU patients (n = 99)	Survival cases (n = 58)	Death cases (n = 41)	*P* value
Male, n (%)	68 (68.7%)	41 (70.7%)	27 (65.9%)	.609
Age, median (range)	69 (62-80)	66(59.5-77.5)	73 (65-81)	.048
Any comorbidity, n (%)				
Hypertension, n (%)	45 (45.5%)	23 (39.7%)	22 (53.7%)	.168
Diabetes, n (%)	18 (18.2%)	11 (19%)	7 (17.1%)	.810
Cardiovascular disease, n (%)	21 (21.2%)	11 (19%)	10 (24.4%)	.515
Pulmonary disease, n (%)	8 (8.1%)	3 (5.2%)	5 (12.2%)	.374
Stroke, n (%)	15 (15.2%)	10 (17.2%)	5 (12.2%)	.490
Malignancy, n (%)	2 (2%)	1 (1.7%)	1 (2.4%)	1.000
Chronic renal insufficiency, n (%)	8 (8.1%)	3 (5.2%)	5 (12.2%)	.374
Chronic hepatitis and cirrhosis, n (%)	10 (10.1%)	8 (13.8%)	2 (4.9%)	.266
Laboratory results				
White blood cell count ( × 10^9^/L)	9.53 (6.15-11.82)	7.11 (5.58-9.82)	11.41 (9.66-15.59)	<.001
Neutrophil count ( × 10^9^/L)	7.48 (4.48-10.48)	5.42 (4.22-8.23)	10.12 (8.36-13.74)	<.001
Neutrophil percentage (%)	84.3 (73.5-89.7)	78.85 (68.98-84.38)	88.7 (86.3-92.9)	<.001
Lymphocyte count ( × 10^9^/L)	0.79 (0.5-1.2)	0.96 (0.59-1.32)	0.6 (0.40-0.96)	.004
Lymphocyte percentage (%)	8.4 (5.5-15.6)	12.45 (8.08-18.28)	5.5 (3.8-8.25)	<.001
Eosinophil count ( × 10^9^/L)	0.03 (0.0-0.13)	0.08 (0.01-0.15)	0.01 (0.0-0.07)	.005
Eosinophil percentage (%)	0.2 (0-1.5)	1.05 (0.18-2.08)	0.03 (0-0.35)	<.001
Basophil count ( × 10^9^/L)	0.02 (0.01-0.04)	0.02 (0.01-0.04)	0.02 (0.01-0.04)	.844
Basophil percentage (%)	0.2 (0.1-0.4)	0.25 (0.2-0.5)	0.2 (0.1-0.3)	.016
Monocyte count ( × 10^9^/L)	0.51 (0.37-0.72)	0.52 (0.40-0.72)	0.51 (0.3-0.72)	.706
Monocyte percentage (%)	6.1 (4.0-8.0)	7.3 (5.65-8.85)	4.6 (3.15-6.15)	<.001
D-dimer (mg/L)	3.46 (1.74-7.24)	2.89 (1.28-4.86)	6.05 (3.18-13.11)	<.001
CRP (mg/L)	35.35 (21.67-57.7)	29.74 (7.86-35.85)	42.2 (35.35-112.6)	<.001
PCT (ng/ml)	0.28 (0.1-0.63)	0.15 (0.07-0.36)	0.60 (0.28-1.49)	<.001
APTT (seconds)	33.6 (30.2-41.3)	33.4 (30.45-38.5)	38.2 (29.85-47.15)	.017

Data are the median (IQR) or n/N (%). *P* values comparing severe cases and moderate cases are from χ^2^, Fisher’s exact test, or Mann–Whitney *U* test. The frequencies of categorical variables were compared using the χ^2^ and Fisher’s exact test as appropriate.

Abbreviations: CRP, C-reactive protein; PCT, procalcitonin; APTT, activated partial thromboplastin time; ICU, intensive care unit; IQR, interquartile range.

With regard to the laboratory results, lower eosinophil counts and percentages were more commonly found in the COVID-19 deaths group compared with the survival group. In the death cases, some of the eosinophil counts even vanished, whereas this was rarely the case in the survival group. Higher WBC counts, neutrophil counts and percentages, D-dimer, CRP, procalcitonin, and APTT were observed in the COVID-19 deaths group, while lower lymphocyte counts and percentages, and basophil and monocyte percentages were observed in this group. However, no significant differences were found in basophil and monocyte counts (Table 3).

### Circulating Eosinophil Count was a Predictive Factor for Death in ICU Patients with COVID-19

The eosinophil predictive value was assessed using the ROC curve, which had an AUC of 0.665 and a cutoff value of 0.04 × 10^9^ for distinguishing survival cases and death cases (Figure 1B). We divided the ICU patients into 2 groups depending on the circulating eosinophil counts: high eosinophil group (≥0.04 × 10^9^/L, n  =  44) and low eosinophil group (<0.04 × 10^9^/L, n  =  55). Multivariable logistic regression analysis showed that eosinophil counts, WBC counts, CRP, albumin, and APTT had a significant association with ICU patients’ mortality, whereas the use of glucocorticoids had no influence on the outcome, and specific results are expressed as ORs and 95% CIs in Table 4. The results indicated that among the influencing factors, albumin had a protective effect, with an OR of 0.832. Remarkably, patients with lower eosinophil counts (<0.04 × 10^9^/L) were more likely to have fatal prognostic outcomes.

**Table 4.** Multivariable Logistic Regression ORs (95%CI) for Death Risk Factors of ICU Patients.

Covariates	Odds ratio	95% CI	*P* value
Eos count <0.04 × 10^9^/L (n = 55)	4.142	(1.093, 18.348)	.044
WBC count	1.267	(1.097, 1.522)	.004
CRP	1.034	(1.012, 1.062)	.006
ALB	0.835	(0.722, 0.948)	.008
APTT	1.105	(1.034, 1.195)	.006
Glucocorticoid	1.331	(0.354, 4.933)	.666

Abbreviations: WBC, white blood cell; CRP, C-reactive protein; ALB, albumin; APTT, activated partial thromboplastin time; OR, odds ratio; CI, confidence interval; ICU, intensive care unit.

## Discussion

Some 2011 patients were admitted to this hospital, more than 100 of these were admitted to the ICU during February 8 to April 15, 2020, and the patients involved in the current study were among these, for whom we obtained the medical records. Our results suggest that eosinophil counts can predict fatal outcomes for COVID-19 patients in the ICU, but contrary to what we hypothesized, eosinophil counts might not have the same predictive role for general ward patients with COVID-19.

Eosinophils are activated in parasitic infections, fungal infections, and viral infections. Previous research has shown that eosinopenia is an independent predictor of death in patients with pneumonia and has the capacity to protect against viral infection,^16,17^ but this protective effect only occurs in some circumstances. Circulating eosinophils normally range below 500 per microliter and could increase 20-fold or more when they exert immune functions.^8^ In patients with asthma, the accumulation of eosinophils in the lungs has risen 10 to 100 times compared with healthy volunteers,^18^ whereas eosinopenia has appeared in patients with COVID-19.^19^ On the basis of the existing data available worldwide, few asthmatic individuals have been vulnerable to COVID-19 infection, which has sparked special interest because asthma is characterized predominantly by eosinophilic inflammation.^20^ This phenomenon could be attributable to the potential virus clearance ability of eosinophils and conventional therapeutics for asthma.^12^ In accord with this observation, eosinopenia was more prominent in patients with severe COVID-19 infection.^19,21,22^ Recently, the protective effect of eosinophils percent on patient survival was found to be varied by race/ethnicity.^23^ Moreover, blood eosinophil counts have correlated with lymphocytes in all patients,^24^ and normalization of eosinophil numbers followed the improvement of clinical status.^25^ On the contrary, a retrospective study included 1035 confirmed COVID-19 patients, one quarter of them were severe cases, reported that eosinopenia was not a useful predictor of mortality.^26^ The studies mentioned above focused on the prognostic indication function of eosinophil levels in patients with COVID-19, but whether eosinophil accumulation in the respiratory system or overall elevation in the human body increases COVID-19 virus resistance has not yet been clarified. An urgent question is also whether the eosinophil level could alter the course of COVID-19 or whether it has only an accompanying role during the infection process.^14^ A recent retrospective study on eosinophils reviewed patients who visited the fever clinics of Shanghai General Hospital from late January to early February, 2020.^27^ This study revealed that the eosinophil count in 12 confirmed patients with COVID-19 from 227 fever clinic outpatients was lower than that in those with other types of pneumonia. In hospitalized patients, eosinophil counts fell below the detection limit in all 12 severe patients, and low eosinophil counts could be related to severe conditions.

The present retrospective study involved 1004 patients within our authority to track their medical records. We found that the primary characteristics of ICU patients included male sex, old age, hypertension, stroke, high WBCs, low neutrophil and lymphocyte counts/ratios, and low eosinophil counts/ratios. These findings are comparable to prior publications.^5-7^ To explore whether eosinophil levels could alter the clinical course of patients with mild COVID-19 infection, we conducted a matching study of 35 ICU and 70 general ward patients, all of whom were first admitted to the general ward of Leishenshan Hospital. Thus, we could track records of their earliest eosinophil level. In a matching study, we balanced several variables that could affect the outcome to reduce interference. After balancing, the risk factors that potentially promote infection from mild to severe status were hypertension, CRP, urea, D-dimer, APTT, procalcitonin, and glucose. It is noteworthy that there was no significant difference between the effect of eosinophil counts <0.02 × 10^9^/L and eosinophil counts ≥0.02 × 10^9^/L on ICU admission after taking other confounders into consideration.

To further define the role of eosinophils in patients with severe COVID-19, we divided 99 ICU patients into 58 survival and 41 death cases. Not surprisingly, old age was the vital risk factor for death compared with other comorbidities. According to previous experience during SARS, patients may benefit from the anti-inflammation property of glucocorticoids, but the administration of glucocorticoids to patients with COVID-19 remains controversial due to their side effects such as osteoporosis and immunocompromisation.^28^ Furthermore, glucocorticoids can reduce EOS release and promote its clearance,^29^ however, in the present study, we failed to discover a significant relevance between the use of glucocorticoids and the death of severe patients with COVID-19 in the ICU. With regard to laboratory data, low eosinophil levels, along with several other markers, such as CRP, were predictors of fatal outcomes in patients with severe COVID-19. In the present study, the eosinophil counts had an AUC of 0.665, and the cutoff value was 0.04 for the prediction of death in ICU patients with COVID-19, whereas others have reported eosinophil counts with an AUC of 0.74 and a cutoff value of 0.015 for the diagnosis of COVID-19 among 109 confirmed cases and 215 other types of pneumonia.^27^ The combination of EOS and other laboratory markers may have better predictive value; however, it is acceptable for an AUC of 0.665 as a single predictor of death in severe patients. Our results were consistent with the findings of Yan et al^30^ who showed that EOS levels were significantly lower in critical patients than in patients with moderate disease progression. The death OR of eosinophil counts below 0.04 × 10^9^/L was 4.142 after multivariable logistic regression analysis in the ICU patients of our study. Yan et al also found that eosinopenia may be the cause of progression to critical disease in patients with COVID-19, which was different from our result showing that after adjusting for confounders that could have affected the outcome, we found that eosinophil counts might not be a predictor of ICU admission. Viral virulence, treatment protocol, and statistical methods may contribute to this difference. Another difference was that their conclusion of EOS predictive value of mortality was made from overall survivors and nonsurvivors, whereas the sample size was larger in our study and the patients in general ward/ICU were separately analyzed in our research.

Our results suggest a predictive value of eosinophils for fatal outcomes in ICU patients. However, a recent study from Columbia University that included 1298 patients with COVID-19 with an asthma prevalence of 12.6% failed to show a significant difference in length of hospital stay, need for intubation, and mortality between asthma and nonasthma patients.^31^ This observation challenges the basic assumption of the protective effect of eosinophils in patients with asthma. Therefore, further prospective studies are urgently required.

## Limitations

The limitation of this study is that owing to its retrospective nature, we could not review the basic eosinophil count for all ICU patients, given that some of them were transferred to the ICU directly from other hospitals or their medical records were not tracked in the general wards.

## Conclusions

Our findings suggest that eosinophil counts might not be predictive of ICU admission but could indicate a death outcome for ICU patients. There was no significant difference in eosinophil counts higher or lower than 0.02 × 10^9^/L on ICU admission among general ward patients, whereas eosinophil counts below 0.04 × 10^9^/L were more likely to have fatal outcomes in ICU patients. Prospective research and more patients are needed to further explore the exact role of eosinophils in COVID-19.

## Supplemental Material

sj-docx-1-jic-10.1177_08850666211037326 - Supplemental material for Predictive Value of Eosinophil Count on COVID-19 Disease Progression and Outcomes, a Retrospective Study of Leishenshan Hospital in Wuhan, China Click here for additional data file. Supplemental material, sj-docx-1-jic-10.1177_08850666211037326 for Predictive Value of Eosinophil Count on COVID-19 Disease Progression and Outcomes, a Retrospective Study of Leishenshan Hospital in Wuhan, China by Wei Xuan, Xuliang Jiang, Lili Huang, Shuting Pan, Caiyang Chen, Xiao Zhang, Hui Zhu, Song Zhang, Weifeng Yu, Zhiyong Peng and Diansan Su in Journal of Intensive Care Medicine
