# Supplementary material for: Predictive Value of Eosinophil Count on COVID-19 Disease Progression and Outcomes, a Retrospective Study of Leishenshan Hospital in Wuhan, China
Source: J Intensive Care Med. 2021 Sep 22;37(3):359–65. doi: 10.1177/08850666211037326 (PMC8986993; doi:10.1177/08850666211037326)
Supplement: sj-docx-1-jic-10.1177_08850666211037326 - Supplemental material for Predictive Value of Eosinophil Count on COVID-19 Disease Progression and Outcomes, a Retrospective Study of Leishenshan Hospital in Wuhan, China [file sj-docx-1-jic-10.1177_08850666211037326.docx]

**Supplementary Table 1. Clinical characteristics after propensity score matching**

| **Characteristics** | **Total**  **(n=105)** | **General ward**  **(n= 70)** | **ICU**  **(n= 35)** | **p value** |
| --- | --- | --- | --- | --- |
| Male, n (%) | 73(69.5%) | 48(68.6%) | 25(71.4%) | 0.881 |
| Age, years | 72 (64-82) | 72.5 (65-81.7) | 71 (63-82.5) | 0.857 |
| **Any comorbidity, n (%)** |  |  |  |  |
| Hypertension, n (%) | 39/105(37.1%) | 22/70(31.1%) | 17/35(48.5%) | 0.033 |
| Coronary heart disease, n (%) | 9/105(8.5%) | 4/70(5.7%) | 5/35(14.2%) | 0.462 |
| Diabetes, n (%) | 10/105(9.5%) | 6/70(8.5%) | 4/35(11.4%) | 0.64 |
| **Laboratory results** |  |  |  |  |
| White blood cell count (×10^9^/L) | 6.0(4.9-8.7) | 5.9(4.9-7.7) | 6.6(4.9-11.5) | 0.082 |
| Neutrophil percentage (%) | 73.5(67.4-81) | 72.5(63.8-79.7) | 75.4(68.9-88.4) | 0.054 |
| Lymphocyte count (×10^9^/L) | 14.9(10.4-20) | 15.3(11.5-19.8) | 14.4(5.2-19.4) | 0.089 |
| AST (U/L) | 26 (17-47) | 24(17-41.9) | 28(18.5-53) | 0.231 |
| CRP (mg/L) | 9.5(2-29.2) | 4.6(1.11-21.8) | 22.7(6.9-104.3) | <0.001 |
| ALB (g/L) | 32.2(29.1-35.3) | 33(30.2-35.3) | 30(27.5-35.1) | 0.054 |
| Urea (mmol/L) | 6.5(4.7-11.3) | 5.8(4.4-8.5) | 9.4(5.5-18.7) | 0.003 |
| Cre (ummol/L) | 73.1(64-98.9) | 71(64.2-95.8) | 74.6(60-178.4) | 0.563 |
| D-dimer(mg/L) | 1.6(0.7-4.4) | 0.9(0.6-3.2) | 3.4(1.5-6.8) | 0.001 |
| APTT(s) | 30.9(27.1-35.8) | 29.9(26.3-33.2) | 34.4(29.2-41.5) | 0.001 |
| PCT(ng/ml) | 0.08(0.04-0.21) | 0.05(0.04-0.10) | 0.32(0.09-1.01) | <0.001 |
| Glu (3.9-6.11) | 5.5(4.8-7.5) | 5.2(4.6-6.2) | 7.2(5.4-10.7) | <0.001 |

Data are median (IQR) or n/N (%). *P* values comparing the group of general ward and ICU patients are from χ² test or Kruskal-Wallis rank sum test . AST: Aspartate transaminase; ALB: Albumin; Cre: Creatinine; APTT: Activated partial thromboplastin time; PCT: Procalcitonin; Glu: Glucose.
